# Active video games: the mediating effect of aerobic fitness on body composition

**DOI:** 10.1186/1479-5868-9-54

**Published:** 2012-05-03

**Authors:** Ralph Maddison, Cliona Ni Mhurchu, Andrew Jull, Harry Prapavessis, Louise S Foley, Yannan Jiang

**Affiliations:** 1 Clinical Trials Research Unit, University of Auckland, Auckland, New Zealand; 2 School of Nursing, University of Auckland, Auckland, New Zealand; 3 School of Kinesiology, Faculty of Medical and Health Sciences, University of Western Ontario, London, ON, Canada; 4 Clinical Trials Research Unit, University of Auckland, Private Bag 92019, Auckland, 1142, New Zealand

**Keywords:** Active video games, Physical activity, Sedentary behavior, Children, Overweight

## Abstract

**Background:**

Increased understanding of why and how physical activity impacts on health outcomes is needed to increase the effectiveness of physical activity interventions. A recent randomized controlled trial of an active video game (PlayStation EyeToy™) intervention showed a statistically significant treatment effect on the primary outcome, change from baseline in body mass index (BMI), which favored the intervention group at 24 weeks. In this short paper we evaluate the mediating effects of the secondary outcomes.

**Objective:**

To identify mediators of the effect of an active video games intervention on body composition.

**Methods:**

Data from a two-arm parallel randomized controlled trial of an active video game intervention (n = 322) were analyzed. The primary outcome was change from baseline in BMI. A priori secondary outcomes were considered as potential mediators of the intervention on BMI, including aerobic fitness (VO_2Max_), time spent in moderate-to-vigorous physical activity (MVPA), and food snacking at 24 weeks.

**Results:**

Only aerobic fitness at 24 weeks met the conditions for mediation, and was a significant mediator of BMI.

**Conclusion:**

Playing active video games can have a positive effect on body composition in overweight or obese children and this effect is most likely mediated through improved aerobic fitness. Future trials should examine other potential mediators related to this type of intervention.

**Trial registration:**

Australian New Zealand Clinical Trials Registry

Website: http://www.anzctr.org.au

Study ID number: ACTRN12607000632493

## Findings

### Introduction

Increased understanding of why and how physical activity impacts on health outcomes is needed to increase the effectiveness of physical activity interventions. A recent randomized controlled trial of an active video game (PlayStation EyeToy™) intervention showed a statistically significant treatment effect on the primary outcome, change from baseline in body mass index (BMI), which favored the intervention group at 24 weeks [1]. Active video games are electronic games that allow players to physically interact (using arm, leg, or whole-body movement) with images onscreen in a variety of activities such as sports (e.g., football, boxing, martial arts) and other activities (e.g., dancing, washing windows).

In this short paper we evaluate the mediating effects of the secondary outcomes. Treatment mediators identify possible mechanisms through which a treatment might achieve its effects. These mechanisms are causal links between treatment and outcome [2].

## Methods

A two-arm parallel randomized controlled trial was conducted in Auckland, New Zealand from February 2008 to January 2010. 322 overweight and obese children aged 10–14 years, who were current users of sedentary video games were randomly assigned to either receive an active video game intervention (n = 160) or no change (control group; n = 162). Full details of recruitment, participant flow through the study and measures have been published elsewhere [1,3].

In brief, participants were recruited through schools and various community locations in Auckland city. Eligible participants were aged 10–14 years, overweight or obese (according to the International Obesity Task Force international cut-offs for child obesity) [4], and owned a PlayStation®2 or 3 gaming console, but no active video games, including EyeToy™ or Nintendo Wii; and played ≧ two hours of video games per week. Participants were excluded if they had contraindications to performing physical activity (such as a medical condition). One child per household was eligible to take part. Dropout from the study was 24% and 17% for the intervention and control groups, respectively.

Intervention participants received an upgrade (hardware and games) of existing gaming technology enabling them to play Sony PlayStation EyeToy™ active video games at home. A selection of active video games (e.g., Play3, Kinetic, Sport, and Dance Factory; Sony) were provided on the basis of being current releases and offering a variety of activity options. Published MET (Metabolic Equivalent) values for these types of games range from 2.0–5.0 [5,6]. Children were encouraged to meet current physical activity recommendations (60 minutes of moderate to vigorous physical activity on most days of the week) [7] by supplementing periods of inactivity with active video game play and substituting periods of traditional non-active video game play with the active version. To ensure the sustainability of the intervention [8] children were sent a package of new active video games at 12 weeks. Active video game play was sustained at higher levels than baseline at both 12 and 24 weeks in intervention participants [1], suggesting this strategy was successful. The control group continued with their normal video game play. On completion of the study, participants in the control group received the active video games upgrade package at no cost.

### Measures

All outcomes were measured at baseline 12 and 24 weeks. Anthropometric data (height and weight) were measured using standard practices [9]. Body fat was assessed using standardized bio-electrical impedance analysis procedures [10] with the ImpediMed DF50 Bioimpedence Monitor (Queensland, Australia). This method has been validated against deuterium dilution and specific prediction equations developed in New Zealand children [11]. Aerobic fitness was assessed using the 20 meter shuttle test [12,13]. Seven day physical activity was measured objectively using accelerometry [14], and daily (for seven days) self-reported snack food consumption was assessed using a participant diary developed and tested in a previous pilot study [8].

The primary outcome was change from baseline in BMI, while percentage body fat was measured as a secondary outcome. A priori secondary outcomes were considered as potential mediators of the intervention effect on BMI and percentage body fat, including aerobic fitness (VO_2Max_), time spent in moderate-to-vigorous physical activity (MVPA), and food snacking at 24 weeks.

In accordance with the recommendations of Kraemer et al [2] for testing mediators of treatment effects in randomized clinical trials, hierarchical regression analyses were conducted on the observed participants’ data with change from baseline to 24 weeks in BMI and percentage body fat as the criterion measures. According to Kraemer and colleagues, a mediator must measure an event or change occurring during treatment, and then it must correlate with treatment choice, hence possibly be a result of treatment, and have either a main or interactive effect on the outcome. Their analytic approach differs conceptually from that of Baron and Kenny [15] in several important ways. According to Kraemer et al. with mediation, demonstration of precedence is required. A mediator occurs during treatment. Similarly, demonstration of correlation is required. They argue that in absence of such criteria, the interpretation of whether a relationship is mediating or moderating is often arbitrary. The analytic model, in contrast to the several linear model proposed by Baron and Kenny, is exactly the same for moderators and mediators. The difference lies in how M (Mediator or Moderator) is defined in terms of time relation to treatment onset and correlation with treatment choice [2].

## Results

All statistical analyses were performed using SAS version 9.1.3 (SAS Institute Inc. Cary NC) and R [16] version 2.10.0. Statistical tests were two-tailed and a 5% significance level maintained throughout the analyses. Only aerobic fitness at 24 weeks met the conditions for mediation, and was a significant mediator of all treatment outcomes (*p* < 0.0001) evaluated in the above models. The level of mediation differed for each response (Table 1). For change in BMI, the observed difference between the two treatment groups reduced from −0.3265 (*p* = 0.01) to −0.2992 (*p* = 0.02), or approximately 8% when aerobic fitness was included. For percentage body fat, the observed group difference shifted from −0.291 (*p* = 0.02) to −0.4136 (*p* = 0.03), or approximately 50%. No statistically significant mediating effects were found for any of the other variables of interest.

**Table 1:** **Treatment effects with mediator variable VO**_**2Max**_

	**Treatment effect**				**Difference in treatment effect**				**Overall mediation**
	Intervention		Control						
Change from baseline	Mean	SE	Mean	SE	Difference in means	SE	Lower 95% C.I.	Upper 95% C.I.	p
BMI (kg/m^2^)									
Main*	0.11	0.11	0.44	0.11	−0.33	0.12	−0.57	−0.08	
VO_2Max_#	0.13	0.11	0.43	0.11	−0.30	0.13	−0.56	−0.04	<.0001
Percentage % Body Fat									
Main*	−0.99	0.29	−0.16	0.30	−0.83	0.36	−1.54	−0.12	
VO_2Max_#	−0.93	0.32	−0.52	0.33	−0.41	0.39	−1.19	0.36	<.0001

Note: * Main effect without mediator.

# Mediator at post-intervention (24 weeks).

## Conclusion

Playing active video games can have a positive effect on body composition in overweight or obese children and this effect is most likely mediated through improved aerobic fitness. Consistent with previous research [17] we found no effect of the intervention on measured physical activity. The lack of effect on measured physical activity might be in part explained by the large variability of physical activity within individuals and low measurement precision of accelerometers to capture non-ambulatory activity. Here aerobic fitness may have served as a proxy measure of increased physical activity. Future trials should examine other potential mediators related to this type of intervention. Such mediators might include attitudes to or preferences for physical activity, or changes in sedentary behaviour. Our study adopted a very pragmatic approach to video game play by allowing the participants as little or as much as they wished; however future studies may find greater utility in prescribing time spent in active gaming for greater effects on aerobic fitness.

## Abbreviations

BMI = Body mass index; MVPA = Moderate-to-vigorous physical activity; VO_2Max_ = Maximal oxygen consumption.

## Competing interests

Sony Computer Entertainment Europe provided the gaming software for the study. The funders and Sony Computer Entertainment Europe played no role in the design, conduct or analysis of the study, nor in the interpretation and reporting of the study findings. The authors declare they have no conflicts of interest.

## Authors’ contributions

RM provided study oversight. LF conducted the research and undertook data collection. YJ performed the statistical analyses. RM, CNM, AJ, AR all designed the research (project conception and development of overall research plan). RM wrote the paper. All authors assisted in interpretation of the analyses and revising the paper. All authors read and approved the final manuscript. RM takes primary responsibility for final content. RM has had full access to all of the data in the study and takes responsibility for the integrity of the data and the accuracy of the data analysis. All authors read and approved the final manuscript.
